# Editorial: Oral health behaviour change

**DOI:** 10.3389/froh.2023.1288512

**Published:** 2023-10-10

**Authors:** Heather Buchanan, Michaela Goodwin, Joanna Goldthorpe, George Kitsaras

**Affiliations:** ^1^ Lifespan & Population Health, School of Medicine, University of Nottingham, Nottingham, United Kingdom; ^2^ Division of Dentistry, University of Manchester, Manchester, United Kingdom; ^3^ NIHR ARC North West Coast, Department of Health Research, Lancaster University, Lancaster, United Kingdom

**Keywords:** behaviour change, dentistry, oral healh, children, health promotion

**Editorial on the Research Topic** Oral health behavior change

Achieving good dental health is a fundamental part of overall well-being. While improvements in dental treatment have been a key focus within dentistry for over 100 years, behaviour and the habits of individuals remains an essential component to good oral health. The recognition of the importance of changing behaviour has led to a fundamental change in perspective within dentistry, concentrating on prevention and individual's behaviour and the impact of these on health outcomes.

Behaviour change and dentistry involve a collaborative effort between key stakeholders, health professionals and the patients they interact with and treat. Key behaviours can include patients adopting effective brushing and flossing habits, maintaining and attending regular dental appointments, adhering to recommended diets (reduced sugar, snacking before bed, etc.), and reducing risk behaviours such as smoking and alcohol consumption. The behaviours of dental professionals are also important to consider; the decisions they make on the prevention and treatment provided, the time taken to engage with their patients and who they treat can all have an impact on individual and population health.

There are several models and theories which are used to explain behaviour change in the context of dental health. The COM-B model provides a framework to understand how the components capability, opportunity and motivation interact and contribute to behaviours which impact on dental health. The COM-B is also used within the Behaviour Change Wheel which can be used to help develop interventions which focus on changing behaviour (1). Aside from the COM-B model, GPS is another approach used to promote and sustain effective behaviour change. GPS stands for: Goal-setting, Planning and Self-Monitoring with these three, simple behaviour change techniques showing promising results in impacting positive oral hygiene changes (2). Finally, MAP again suggests that a smaller number of effective techniques, in this case: Motivation, Action and Prompts can be successful in changing behaviours with most of this work having taken place outside of dentistry (3). Most of the studies in this Research Topic have employed these theories and models (as well as others, see Social Learning Theory) as a basis for their research, which is encouraging. There is increasing evidence to suggest that behavioural interventions based on theory are more effective than those that are not (4). Theories enhance our understanding of complex phenomena, such as human behaviours, especially the circumstances under which behaviours occur, the drivers of behaviours, and how best to address them. Theories also provide support for a more in-depth understanding of what works (and what doesn't work) in interventions.

The four papers included in this Research Topic showcase a number of different topics, settings and methodologies. Indeed, it is exciting to see so many approaches within behaviour change in oral health! It's also encouraging to see the extent which this work is collaborative and truly multidisciplinary; which is important for the advancement of behavioural research in this context (5). For example, health psychologists, public health specialists and different members of the dental team (across many different specialities) have come together to produce these varied research studies. The different perspectives and expertise have contributed to the quality and breadth of the papers which are included in this edition.

Kristensen et al. have brought together the research on psychologically informed oral health interventions for pregnant women with type-2 diabetes and the extent to which the interventions map on to COM-B. They also considered the outcomes that were targeted within these interventions. Findings from their scoping review indicate that oral health is indeed recognised aspect of pregnancy and type-2 diabetes, which is encouraging as good oral health in gestational diabetes is vital. Oral-health related knowledge was the most targeted concept with most studies using an educational approach in interventions. Importantly, these findings are being used by the authors to develop the first intervention in the UK to target oral health in gestational diabetes in pregnant women.

Demonstrating the breadth of the topics and methodologies in this Research Topic, Kitsaras and Goodwin have analysed how oral hygiene and risk behaviours are portrayed in animated films. Characters in movies may have a great influence on child viewers, and as such portrayal of negative and positive oral health behaviours may be important for developing habits in young audiences. In a systematic approach, they coded oral hygiene and oral health risk behaviours in the top 30 highest grossing animated films. Unfortunately, (though perhaps not surprisingly) the vast majority (93%) of the behaviours were risk related. This evidence clearly demonstrates that children are still being exposed to behaviours that do not reinforce positive oral health behaviours. However, the authors note that a positive step forward from this study, would be a consensus on how to portray positive oral health behaviours across media, including positive portrayal of the dental team.

There is growing recognition of the significant global health burden of periodontal disease. Indeed, a recent widely cited report based on six Western European countries, found that more effective prevention of periodontal disease could save billions in health care costs and lead to healthier lives (6). Therefore, it is timely that Chan et al. have conducted a narrative review on theory-based behaviour change interventions to specifically improve periodontal health. They found that four theory-based interventions could improve periodontal health but comparing these was difficult due to the differences in study design and theories (and constructs) involved. They recommend standardisation of naming and reporting of theories and frameworks, as well as periodontal parameters to conduct meaningful comparisons.

Finally, Rutter et al. have focused on the role of the dental practitioner in oral health behaviour change, more specifically newly qualified UK dentists. In their qualitative study they explored the barriers and facilitators to these early career dentists conducting conversations about oral health with parents and caregivers and their children. Ensuring these newly qualified dental professionals have the motivation and skills to have these conversations so early in their career, could be transformative in terms of preventive oral health behaviour for children and their families. From their analyses and findings, they make recommendations about what is needed to help develop training including the need for a multifaceted approach.

It is an exciting time for behaviour change in oral health, as evidenced by the diverse articles highlighted in this Research Topic. The authors have considered carefully how to take forward their findings, and thus there are some interesting and varied pointers towards future research. We hope you enjoy reading them.
